# Adrenal Steroid Metabolism and Blood Pressure in 5- to 7-Year-Old Children Born Preterm as Compared to Peers Born at Term

**DOI:** 10.3389/fped.2021.754989

**Published:** 2021-11-30

**Authors:** Eva Landmann, Markus Brugger, Verena Blank, Stefan A. Wudy, Michaela Hartmann, Konstantin Strauch, Silvia Rudloff

**Affiliations:** ^1^Department of Pediatric Hematology and Oncology, Center of Child and Adolescent Medicine, Justus Liebig University Giessen, Giessen, Germany; ^2^Institute of Medical Biometry and Epidemiology, Philipps-University Marburg, Marburg, Germany; ^3^Institute of Genetic Epidemiology, Helmholtz Zentrum München - German Research Center for Environmental Health, Neuherberg, Germany; ^4^Institute of Medical Information Processing, Biometry and Epidemiology - IBE, Chair of Genetic Epidemiology, LMU Munich, Munich, Germany; ^5^Institute of Medical Biostatistics, Epidemiology and Informatics (IMBEI), University Medical Center, Johannes Gutenberg University, Mainz, Germany; ^6^Department of Neuropediatrics, Center of Child and Adolescent Medicine, Justus Liebig University Giessen, Giessen, Germany; ^7^Department of Pediatrics and Neonatology, Justus Liebig University Giessen, Giessen, Germany; ^8^Steroid Research and Mass Spectrometry Unit, Division of Pediatric Endocrinology and Diabetology, Center of Child and Adolescent Medicine, Justus-Liebig-University Giessen, Giessen, Germany; ^9^Institute of Nutritional Science, Justus Liebig University Giessen, Giessen, Germany

**Keywords:** blood pressure, preterm, prepubertal, steroid metabolism, preterm birth, cortisol, adrenal steroid, developmental origin of adult disease

## Abstract

**Background:** Previous studies indicated preterm birth to be a risk factor for hypertension in adolescence and adulthood. However, studies in children investigating the underlying mechanisms are scarce.

**Objective:** We hypothesized children born preterm to have higher excretion of cortisol and/or androgen metabolites per day concomitantly with higher blood pressure as compared to peers born at term. We thus aimed to compare urinary steroid profiles and blood pressure between 5- to 7-year-old children born preterm and peers born at term. Furthermore, aldosterone precursor excretion per day was compared between both groups.

**Methods:** Blood pressure was measured in 236 children (preterms *n* = 116; gestational age 29.8 ± 2.6 (30; 24–33) weeks [mean ± standard deviation (median; range)]) using an automatic oscillometric device. Urinary steroid profiles were determined in 24-h urine samples (preterms *n* = 109; terms *n* = 113) using gas chromatographic-mass spectrometric analysis. To assess excretion of cortisol and androgen metabolites per day, major cortisol and androgen metabolites were summed, respectively. To assess aldosterone excretion per day tetrahydrocorticosterone, 5α-tetrahydrocorticosterone, and tetrahydro-11-deydrocorticosterone were summed.

**Results:** Multiple regression analyses showed prematurity to be associated with systolic but not with diastolic blood pressure. When adjusted for potential confounders (prematurity, gender, age at day of examination, being born small for gestational age, breastfeeding, accelerated weight gain during infancy, family history of cardiovascular disease, parental hypertension, and body mass index) prematurity was shown to be associated with an increase in systolic blood pressure by 2.87 mmHg (95% confidence interval 0.48–5.27; *p* = 0.02). Cortisol, androgen metabolite, and aldosterone precursor excretion per day were not higher in individuals born preterm. In contrast to our hypothesis, multiple regression analysis showed prematurity to independently decrease cortisol and aldosterone precursor excretion per day (*p* < 0.001 and 0.04, respectively).

**Conclusion:** This study provides further evidence for systolic blood pressure to be higher after preterm birth as early as at the age of 5 to 7 years. However, this seems not to be explained by elevated excretion of cortisol and/or androgen metabolites.

## Introduction

Several studies indicate higher blood pressure (BP) values in adolescents and in adults born preterm (1–9). Data on blood pressure before adolescence in children born preterm as compared to children born at term are comparatively scarce. Two groups (10, 11) described higher BP in toddlers born preterm as compared to toddlers born at term. Some reports on BP in school-age children born preterm had been restricted to specific groups, such as individuals born small for gestational age (SGA) (12) or born at an extremely low gestational age (13). Only recently, data on blood pressure in 5- to 7-year-old children born preterm as compared to peers born at term were published (14–17). Further data are needed to elucidate whether, already at early school-age, individuals born preterm exhibit differences in BP as compared to peers born at term as reported in most—but not all—of these recent studies.

The underlying mechanisms that could lead to higher blood pressure as a sequela of preterm birth are not fully understood.

Increased BP after preterm birth might derive from altered hypothalamus-pituitary-adrenal (HPA) axis function. A stressful perinatal period—as it is experienced by children born preterm—might modify HPA axis functions (18, 19) with subsequent alterations in cortisol metabolism.

The aim of the present study was to compare (a) adrenal steroid metabolism and (b) BP between 5 and 7-year-old children born at a gestational age of 33 weeks or below (preterm group) and a group of children born at a gestational age between 37 and 41 weeks (term group). We hypothesized children born preterm to have higher excretion of cortisol and/or androgen than their peers born at term. We also hypothesized children born preterm to have higher BP as compared to children born at term.

To comprehensively describe adrenal steroid metabolism, we further measured the excretion of dihydroepiandrosterone (DHEA) and of aldosterone precursors per day [as urinary excretion of mineralocorticoids per day (ME/d)] in children born preterm and in children born at term by performing urinary steroid profiles in 24-h urine samples using gas chromatographic-mass spectrometric analysis.

## Subjects and Methods

### Study Population and Ethics

All children who participated in the study were between 5 and 7 years old at the day of examination. Preterm children of a gestational age of 33 weeks or below were included in the study (preterm group). The control group comprised children of a gestational age between 37 and 41 weeks (term group). Children were recruited from the region of Hesse, Germany. We aimed to motivate as many parents of children born preterm as possible to participate in the study. Therefore, we chose several ways to recruit children born preterm. In Germany, one way to comprehensively circulate information to parents is the obligatory health examination before school entry, which is performed by the local public health services. The health examination is obligatory for all children, including mentally and/or physically handicapped children. All public health services within the radius of 120 km distributed information on the study to parents of children born preterm when they showed up for their obligatory health examination. In addition, pediatric offices within the same circumference were asked to distribute information on the study to the parents of children born preterm. Children born at term were also recruited through the obligatory health examinations before school entry as well as via newspaper advertisements. Recruitment and characteristics of both groups have been previously described in detail (20).

Exclusion criteria for all children were: type-1 diabetes, chromosomal abnormalities, major disability (gross motor function classification system > II) (21), chronic illness, and systemic corticosteroid therapy. All children had to be prepubertal, i.e., each child had to be Tanner stage I as ascertained by the study physician. Gestational age was taken from the hospital birth records. Detailed data concerning pregnancy, perinatal history, previous medical history, nutrition, growth, and weight gain were taken from the documents of the obligatory well-child visits and were complemented by information obtained from medical records. Additional information on the child's history as well as on family history was obtained by a structured interview at the day of examination.

Being born SGA was defined as a birth weight below the 10th percentile according to the percentiles of Voigt (22) for German newborns. Accelerated weight gain during infancy was defined as an increase in the percentile values of more than 25 percentiles between birth and the first birthday. A history of parental hypertension was ascertained if at least one parent reported to receive antihypertensive medication. A family history of cardiovascular disease was ascertained if at least one parent or grandparent had suffered coronary heart disease and/or myocardial infarction and/or stroke.

The study was approved by the local research ethics committee and written parental consent was obtained.

### Anthropometry

Body weight and length were measured as recommended by Stolzenberg et al. (23) and body mass index (BMI) was calculated (24).

### BP Measurements

After a 10-min rest, with the subject in a sitting position, BP was measured from the right upper arm three times with an interval of at least two min using an automatic device (Welch Allyn®, 52000 Series, Akanetateles Falls, New York, NY, USA). The measurements were taken by a trained study nurse. The cuff was chosen depending on the size of the child's upper arm as recommended by the American Academy of Pediatrics (25). The arithmetic means of three measurements for systolic and diastolic BP (SBP and DBP) were included in the analysis.

### Urinary Adrenal Steroid Profiles in 24-H Urine Samples by Gas Chromatography-Mass Spectrometry

The study participants collected a 24-h urine sample for quantification of adrenal steroid hormone metabolites. The 24-h urine sample was collected within two weeks after the study visit.

Urinary steroid profiles were analyzed using quantitative data that were generated by gas chromatography-mass spectrometry (Agilent Technologies 6890) analysis as described previously (26, 27).

To assess urinary cortisol excretion per day (CE/d), the seven major urinary glucocorticoid metabolites, i.e., tetrahydrocortisol, 5α-tetrahydrocortisol, α-cortol, β-cortol, tetrahydrocortisone, α-cortolone, and β-cortolone were summed (27).

To assess urinary overall androgen metabolite excretion of the adrenals per day (AE/d), the sum of androsterone, etiocholanolone, 5-androstene-3β, 17α-diol, 5-androstene-3β,17β-diol, DHEA, 16α-hydroxy-DHEA, and androstenetriol-16α was calculated (26).

To assess aldosterone precursor excretion per day (ME/d), tetrahydrocorticosterone, 5α-tetrahydrocorticosterone, and tetrahydro-11-deydrocorticosterone were summed.

Absolute amounts of metabolites, sums, and ratios calculated were divided by the absolute amount of 24-h urinary creatinine excretion. For comparisons between term and preterm born children, these values were additionally divided by BMI or body surface area (BSA). Furthermore, these comparisons were done separately for boys and girls, because reference values for urinary steroid metabolites differ between both genders (26, 27).

### Statistical Methods

For continuous data, two sample *t*-tests were applied in case of a normal distribution, Mann-Whitney U tests when the values were not distributed normally. Because data on urinary steroid profiles can contain an appreciable number of zero values, we applied a two-part permutation test (28). Dichotomous variables were compared using Fisher's exact test.

Since being born SGA is known to be associated with the development of metabolic and cardiovascular disease later in life, all analyses were also performed excluding children with SGA.

Further, multiple regression analyses were calculated to assess the influence of prematurity on blood pressure and urinary steroid profiles. The regressions were performed as follows.

(1) Multiple regression analyses were performed to describe the influence of prematurity on SBP and DBP. Initially, the following variables were included in the full model: prematurity, gender, age at day of examination, SGA, breastfeeding, accelerated weight gain during infancy, family history of cardiovascular disease, parental hypertension, and BMI.

(2) Multiple regression analyses were calculated to assess the influence of prematurity on CE/d, on AE/d, on ME/d, and on DHEA/d. Log-transformation was performed for the outcomes CE/d, AE/d, and ME/d, but not for DHEA/d, for which regression diagnostics showed that the residuals of the full model with DHEA/d as log-transformed outcome variable were not normally distributed. Therefore, we applied a Box-Cox transformation (29) to DHEA/d in order to obtain normally distributed residuals. In addition to the variable prematurity, the variables gender, age at examination, and being born SGA were included as potential confounders in the full model. In a secondary analysis, either BSA or BMI was added to the full model since body fat is known to be an endocrine organ.

(3) Backward elimination based on Akaike's information criterion (AIC) was chosen as the model selection algorithm to identify the best-fitting models based on the full models of (1) and (2). For the model selections regarding urinary steroid profiles in (2), both BSA and BMI were added to the full model.

Prematurity, gender, SGA, breastfeeding, accelerated weight gain during infancy, family history of cardiovascular disease, and parental hypertension were included as dichotomous variables in the regression models. Age at day of examination, BMI, and BSA were included as continuous variables in the regression models.

A *p*-value <0.05 was considered statistically significant. Statistical analyses were performed with statistical software package R, version 4.0.3 (30).

## Results

### Baseline Characteristics and Anthropometric Data

A total of 236 children were included in the study. Out of these, 116 children were born preterm, i.e., of a gestational age of 24 to 33 weeks, and 120 children were born at term (37 to 41 weeks of gestational age). Baseline characteristics and anthropometric data of both groups are summarized in Table 1.

**Table 1:** Baseline characteristics of children born preterm and children born at term.

	**Preterm group (*n* = 116)**	**Term group (*n* = 120)**	***p*-value**	**OR/MD^b^**	**95% CI**
Boys/Girls	75/41	67/53	0.18	1.44	0.85; 2.50
Age (years)^a^	6.6 ± 0.8	6.6 ± 0.9	0.98	0.003	−0.21; 0.22
Gestational age (weeks)^a^	29.8 ± 2.6	39.4 ± 1.2	<0.001	−9.67	−10.20; −9.16
Birth weight (grams)^a^	1,434 ± 470	3,486 ± 484	<0.001	−2,052	−2,174; −1,930
SGA	9/116	8/120	0.80	1.18	0.39; 3.65
Body weight (kg)^a^	21.8 ± 4.7	24.3 ± 5.2	<0.001	−2.58	−3.85; −1.30
Body height (cm)^a^	120 ± 8	123 ± 8	<0.001	−3.69	−5.66; −1.73
BMI (kg/m^2^)^a^	15.1 ± 1.9	15.9 ± 2.0	0.003	−0.79	−1.29; −0.28
Family history of cardiovascular disease	19/116	18/120	0.86	1.11	0.54; 2.31
Parental hypertension	23/116	10/120	0.01	0.37	0.15; 0.86
BMI father (kg/m^2^)^a^	26.9 ± 3.9	26.5 ± 3.4	0.48	0.34	−0.61; 1.29
BMI mother (kg/m^2^)^a^	26.3 ± 6.7	25.3 ± 4.7	0.20	0.99	−0.52; 2.49

a *Presented as means and standard deviation*.

b *For dichotomous variables, the odds ratio (OR) is given as effect estimate, whereas the mean difference (MD) is provided for quantitative variables*.

### Blood Pressure

SBP and DBP were on average higher in preterm-born children than in term-born children, although the differences were not statistically significant. However, after excluding SGA, a statistically significant difference was found for DBP between the two groups (Table 2).

**Table 2:** Raw blood pressure values in children born preterm and children born at term.

		**Preterm group (*n* = 116)**	**Term group (*n* = 120)**	**Difference [95% CI]**	***p* value**
Systolic blood pressure [mmHg]^a^	All children	107.6 ± 8.2	105.8 ± 7.7	1.82 [−0.23; 3.87]	0.08
	SGA excluded	107.6 ± 8.0	105.8 ± 7.8	1.86 [−0.25; 3.98]	0.08
Diastolic blood pressure [mmHg]^a^	All children	66.5 ± 6.2	65.0 ± 5.3	1.47 [0; 2.94]	0.05
	SGA excluded	66.6 ± 6.2	65.1 ± 5.2	1.58 [0.06; 3.11]	0.04

a *Presented as means and standard deviation*.

Multiple regression analysis (*n* = 221; with *n* = 109 and *n* = 112 for preterm and term born children, respectively) showed prematurity and BMI to be associated with higher SBP (effect estimates: prematurity 2.87, 95% confidence interval (CI) 0.48–5.27; *p* = 0.02; BMI 1.31, CI 0.77–1.85; *p* < 0.001, adjusted r-squared 0.095). Thus, the variable “being born prematurely” increased SBP by 2.87 mmHg when corrected for potential confounders. After backward elimination, only the variables prematurity and BMI remained in the model (effect estimate for prematurity 3.01, CI 0.97–5.05; *p* = 0.004, and 1.32, CI 0.81–1.83; *p* < 0.001 for BMI; adjusted r-squared 0.11).

For DBP as the dependent variable, no significant association with any predictor was found when performing multiple regression analyses.

### Adrenal Steroid Profiling in 24-H Urine Samples

24-h urine samples were available from a total of 222 children. Table 3 summarizes data characterizing the adrenal steroid metabolism.

**Table 3:** Adrenal steroid metabolites in children born preterm and children born at term.

		**Boys**			**Girls**		
		**Preterm group (*n* = 70)**	**Term group (*n* = 64)**	***P^b^***	**Preterm group (*n* = 39)**	**Term group (*n* = 49)**	***P^b^***
CE/d^a^	SGA included	3.71 (0.96–9.80)	3.75 (1.23–18.89)	0.21	3.28 (0.98–14.46)	4.81 (1.17–14.44)	0.02
	SGA excluded^†^	3.79 (0.96–9.80)	3.75 (1.23–18.89)	0.20	3.28 (0.98–14.46)	4.84 (1.17–14.44)	0.03
ME/d^a^	SGA included	0.44 (0.09–1.24)	0.46 (0.10–2.66)	0.59	0.42 (0.12–2.09)	0.51 (0.13–1.69)	0.19
	SGA excluded^†^	0.45 (0.09–1.24)	0.46 (0.10–2.66)	0.55	0.42 (0.12–2.09)	0.52 (0.12–1.69)	0.15
AE/d^a^	SGA included	0.24 (0.02–1.76)	0.24 (0.06–1.51)	0.85	0.25 (0.05–1.59)	0.38 (0.04–1.41)	0.14
	SGA excluded^†^	0.25 (0.02–1.76)	0.25 (0.06–1.51)	0.99	0.25 (0.05–1.59)	0.38 (0.04–1.41)	0.15
DHEA/d^a^	SGA included	0.01 (0.00–0.50)	0.01 (0.00–0.11)	0.71	0.01 (0.00–0.47)	0.02 (0.00–0.11)	0.70
	SGA excluded^†^	0.01 (0.00–0.50)	0.01 (0.00–0.11)	0.71	0.01 (0.00–0.47)	0.02 (0.00–0.09)	0.65

a *Values are divided by body mass index and are given in μg/mg urinary creatinine secretion per day. Data are presented as median and range*.

b *P value of the two-part permutation test*.

† *When SGA excluded in boys: preterm group n = 65 and term group n = 60; when SGA excluded in girls: preterm group n = 35 and term group n = 45*.

#### CE/d

In boys, no significant differences in CE/d were found between those born preterm and those born at term, regardless of whether the values were divided by BMI or BSA. In girls, higher CE/d was shown in those born at term as compared to girls born preterm, regardless of whether the values were divided by BMI or BSA. For all children (data not shown), higher values were shown for children born at term as compared to those born preterm. The differences described remained significant after exclusion of children born SGA. Multiple regression analysis (*n* = 221) showed the variable prematurity to independently decrease urinary CE/d (*p* < 0.001). Moreover, when BMI or BSA were additionally included in the model, the variable prematurity still significantly decreased urinary CE/d (*p* < 0.001 and *p* = 0.006, respectively). The optimal model according to the AIC included both prematurity and BSA (*p* = 0.004 for prematurity and *p* = 0.01 for BSA; adjusted r-squared 0.08).

#### ME/d

In boys, in girls, and in all children (data not shown), no significant differences with respect to ME/d were found between children born preterm and children born at term, regardless of whether the values were divided by BMI or BSA or children born SGA were excluded from the analyses.

Multiple regression analyses showed the variable prematurity to significantly decrease ME/d (*p* = 0.04). This effect was slightly lower when either BMI (*p* = 0.06) or BSA (*p* = 0.09) were added to the model. After backward elimination, only prematurity remained in the model (*p* = 0.03 and adjusted r-squared 0.016).

#### AE/d and DHEA/d

In all children, whether analyzed separately as boys and girls or independent of their gender (data not shown), no significant differences with respect to AE/d or DHEA/d were found between children born preterm and children born at term, regardless of whether the values were divided by BMI or BSA or children born SGA were excluded from the analyses. Multiple regression analyses also showed no influence of the variable prematurity on AE/d or DHEA/d.

## Discussion

We compared BP and urinary steroid profiles between 5 and 7-year-old children born preterm and peers born at term.

### Study Population

We defined children of a gestational age of <34 completed weeks as our group of children born preterm since we aimed to include an extended spectrum of children born preterm, but also to have a group of preterm children that clearly differed from children born at term. We therefore included children born extremely and moderately preterm. Kramer et al. (31) defined moderate preterm birth as being born at a gestational age of 32 and 33 completed gestational weeks. A gestational age of 34 weeks and more has been suggested to define the cutoff for “late-preterm” (32, 33). The same cutoff has been used by Sipola-Leppänen et al. (5) who reported increased BP in adolescent girls born preterm.

We chose the narrow age span of 5–7 years to investigate BP and steroid metabolism between individuals born preterm and individuals born at term in order to minimize age-related variability and to thus enable better comparability of the values obtained. Further, we examined children younger than 8 years in order to study children that were unambiguously prepubertal and in whom adrenarche, i.e., adrenal androgen secretion, had not started yet.

### Blood Pressure

In our group of 5-to 7-year-old children, prematurity was shown to be associated with an increase in SBP by 2.87 mmHg when adjusted for potential confounders. Differences in DBP, however, can mostly be explained by confounders and hence are not directly related to prematurity.

Our findings are in line with recent studies in late preschool and early school age children born preterm that report higher blood pressure readings as compared to age-matched children born at term (14, 17) and/or an increased prevalence of hypertension in preterm born children (15).

In conjunction with studies reporting higher BP in toddlers born preterm (10, 11), it may be assumed that in children born preterm, BP starts to deviate from normal BP at a young age and continues to deviate throughout childhood and adolescence. This is in line with a report on an inverse relationship between gestational age and BP in 9–12-year-old children (34).

The underlying mechanisms of increased BP values in individuals born preterm, however, have not yet been clarified. Several associations have been described: Singhal et al. (35) reported breast milk consumption to be associated with lower BP in adolescents born prematurely. Vohr et al. (36) reported higher weight gain within the first three years of life to be associated with higher BP in adolescents born preterm, while Bonamy et al. (10) described an inverse association between post-neonatal weight gain and BP in children born preterm at 2.5 years of age.

In our group of children born preterm, multiple regression analyses showed neither breastfeeding nor accelerated weight gain during infancy to be associated with SBP at the age of 5 to 7 years. Furthermore, multiple regression analyses did not show associations between SBP and the variables being born SGA, gender, cardiovascular disease in the family, and parental hypertension, thus supporting the hypothesis that high SBP in adolescents and adults born preterm has a perinatal origin, primarily linked to low gestational age.

### Adrenal Steroid Profiling in 24-H Urine Samples

We hypothesized differences in BP between individuals born preterm and individuals born at term to be explained by differences in cortisol metabolism: A stressful perinatal period as it is experienced by children born preterm might result in programming of the HPA axis (18, 19) resulting in increased secretion of cortisol and its metabolites. Subclinical hypercortisolism is associated with increased BP (37, 38) and might thus mediate the link between prematurity and higher BP values in later life. Positive correlations between BP and serum cortisol levels measured at the age of 2 years were reported in a subgroup of boys with very low birthweight (39).

#### Urinary Cortisol Excretion in 24-H Urine Samples

In contrast to our hypothesis, prematurity was not associated with higher CE/d, but with significantly lower CE/d in preterms as compared to their peers born at term.

Recently, other groups also studied cortisol in former preterm children compared to children born at term of a similar age group as in our study, i.e., 6–7 years: Watterberg et al. (40) also hypothesized preterm-born children to have increased cortisol compared to term-born children. They measured salivary cortisol levels four times during a study visit and in addition three specimen were obtained at home to analyze diurnal patterns. In contrast to their hypothesis, they reported similar cortisol levels in preterm-born children compared to term-born children but with a blunted morning cortisol.

Brummelte et al. (41) measured cortisol levels in three saliva samples collected during a study visit in which children underwent cognitive testing. In addition, cortisol was measured in eight saliva samples collected at home over two consecutive days. They described similar cortisol profiles in preterm and full-term children except for cortisol levels measured at bedtime. Bedtime cortisol levels were higher in the preterm group.

Cortisol levels were also studied in preterm-born children that were younger or older than 6–7 years. Studies performed in younger children had indicated elevated cortisol secretion (42) in individuals born preterm. At the age of 18 months corrected for gestational age, Grunau et al. (42) measured higher cortisol concentrations in a cohort of children born at low gestational age as compared to peers born at term. Studies in preterm-born children studied at an older age than the children examined in our study also hint at higher cortisol secretion: Buske-Kirschbaum et al. (43) showed higher cortisol concentrations after awakening in 18 preterm children aged 8–14 years as compared to 18 control children. In morning urine samples obtained from 36 children aged 8–11 years who were born preterm, Gohlke et al. (44) reported higher cortisol, cortisol metabolites and adrenal androgens as compared to 36 peers born at term.

Taken together, data on cortisol secretion in children born preterm as compared to peers born at term are inconsistent. At least, data obtained from 6–7-year-old children, do not point to higher cortisol secretion in preterm-born children as compared to term-born children. However, studies performed in older children rather indicate increased cortisol secretion in former preterms.

Differences with respect to the age groups studied, the specific study populations, the study designs as well as the methods applied for cortisol measurements do not allow unconfined comparisons between all those studies. For example, the age ranges of 8–11 years (44) or even 8–14 years (43) encompass adrenarche and puberty which might influence cortisol metabolism. Moreover, different methods for measuring cortisol can be used. Measurements in saliva reflect cortisol production at selected points in time (40–43). Urinary steroid profiling as applied by Gohlke et al. (44) in morning urine samples and in our study in 24-h urinary specimens, however, represent integrated measurements, i.e., excretion rates of cortisol and thus allow for the assessment of hormonal production rates.

Data obtained from adults are inconsistent, i.e., in a prospective follow-up study including 147 30-year-olds born at term and 311 subjects of the same age group born preterm, Dalziel et al. (1) could not show an association between prematurity and early morning serum cortisol levels. In addition—and in accordance with our results—preterm birth was shown to be associated with increased SBP. In a comparatively small group, Walker et al. (45) showed lower total urinary cortisol metabolite excretion in 22- to 25-year-old women who were born preterm and appropriate for gestational age as compared to peers born at term.

#### Urinary Androgen Metabolite, DHEA and Aldosterone Precursor Secretion

Adrenal hyperandrogenism and higher ME/d might also explain higher BP values in children born preterm. We also studied AE/d, DHEA/d separately, and ME/d in our group.

In our study, prematurity was not associated with higher AE/d or DHEA/d. In contrast to our hypothesis, prematurity was even associated with lower ME/d.

A few studies also compared adrenal androgens between individuals born preterm and born at term. In contrast to our study, their results rather indicate higher adrenal androgen secretion. Meuwese et al. (46) measured higher DHEA concentrations in serum from young adults born preterm. Watterberg et al. (40) measured salivary DHEA in 5–7-year-old children born preterm as compared to children born at term. In morning urine samples obtained from 36 children aged 8–11 years who were born preterm, Gohlke et al. (44) reported higher cortisol and cortisol metabolites and adrenal androgens as compared to 36 peers born at term. Again, the study population as well as the method applied, do not allow an unconfined comparison with our results. Firstly, the chosen age range of 8–11 years is an age that encompasses adrenarche and enhanced adrenarche in the group of children born preterm may influence the results. Secondly, although urinary steroid metabolites were quantified using gas chromatography-mass spectrometry, morning urine samples reflect adrenal steroid metabolism less precisely than 24-h urine samples.

We did not expect CE/d and ME/d to be even lower in children born preterm than in children born at term. The underlying biological mechanism remains unclear. One might speculate that the adrenal cortex remains smaller after preterm birth just as it had been shown for kidney size (47). A reduced adrenal cortex area might affect adrenal steroid metabolism.

Also, one might speculate that prolonged exposure to stress as it is often experienced by the preterm neonate during the perinatal period might lead to a hypoactive rather than a hyperactive HPA axis function. This hypothesis is supported by the finding of lower HPA axis responses in an adult preterm cohort during stress-test (48). Consistent with the results of that study, our results argue against a major role of HPA axis and adrenal steroid metabolism in explaining increased BP values found in individuals born preterm. We speculate that other mechanisms, e.g., reduced nephron endowment [reviewed in (49) and (50)] or variations in capillary density as recently described by Lewandowski et al. (51) underlie higher BP values measured in individuals born preterm. Exposure to hyperoxia and oxidative stress in the neonatal period might also contribute to the increased risk of increased BP in neonates born preterm as reviewed in (52).

### Strengths and Limitations of the Study

Strengths of the current study include the completeness of data on BP measurements and comprehensiveness of data on urinary adrenal steroid profiles. Furthermore, urinary adrenal steroid profiling in 24-h urinary specimens represent integrated measurements, i.e., excretion rates, and thus allow for the assessment of hormonal production rates. This is in contrast to most studies revealing determinations of steroid concentration in blood or saliva at selected time points only. A further strength of the study is the comparably large group of children born preterm. To the best of our knowledge, urinary steroid profiles have not been studied in larger groups of preterm-born children of that age group. Moreover, we consider the choice of a narrow age range in prepubertal children as an advantage of the study. Limitations of the study are the cross-sectional study design and the fact that neonatal data have not been collected prospectively.

### Summary and Perspective

Our data indicate that—as early as at the age of 5 to 7 years—individuals born preterm seem to be at increased risk for exhibiting higher SBP as compared to peers born at term. This might have implications for their cardiovascular health later in life.

However, our results do not support the hypothesis that elevations in adrenal steroid metabolites explain higher SBP values found in the group of children born preterm. We speculate that other mechanisms or a combination thereof contribute to the increased hypertension risk in individuals born preterm. The identification of possible contributors remains crucial since it might enable the implementation of pre- and postnatal strategies to prevent long-term cardiovascular morbidity.

## Data Availability Statement

The dataset presented in this article is not readily available for reasons of data protection. Requests to access the dataset should be directed to the corresponding author.

## Ethics Statement

This studies involving human participants were reviewed and approved by Ethic Committee of the Faculty of Medicine, Justus-Liebig-University Giessen, Giessen, Germany. Written informed consent to participate in this study was provided by the participants' legal guardian/next of kin.

## Author Contributions

EL conceptualized and designed the study and drafted the initial manuscript. MB and KS carried out the statistical analyses and contributed to the draft of the manuscript. VB carried out the initial data collection with regard to medical examinations and initial analyses. As part of her doctoral thesis she contributed to the initial draft of the manuscript. SW and MH signed responsible for the analyses of steroid metabolites in urine including the interpretation of the data. SR was a co-author of the funding proposal, she thus substantially contributed to the study design, coordination of the project, and critically revised and reviewed the manuscript. All authors contributed to the article and approved the submitted version.

## Funding

This study was supported by the German Research Foundation (DFG grant no. LA 2428/1-1).

## Conflict of Interest

The authors declare that the research was conducted in the absence of any commercial or financial relationships that could be construed as a potential conflict of interest.

## Publisher's Note

All claims expressed in this article are solely those of the authors and do not necessarily represent those of their affiliated organizations, or those of the publisher, the editors and the reviewers. Any product that may be evaluated in this article, or claim that may be made by its manufacturer, is not guaranteed or endorsed by the publisher.

## Abbreviations

AE/d: urinary excretion of androgen metabolites per day
BMI: body mass index
BP: blood pressure
BSA: body surface area
CE/d: urinary excretion of cortisol per day
DBP: diastolic blood pressure
DHEA: dehydroepiandrosterone
HPA: hypothalamic-pituitary-adrenal axis
ME/d: urinary excretion of mineralocorticoids per day
OR: odds ratio
SBP: systolic blood pressure
SGA: small for gestational age.
